# Advances for the treatment of lower extremity arterial disease associated with diabetes mellitus

**DOI:** 10.3389/fmolb.2022.929718

**Published:** 2022-08-17

**Authors:** Yang Pan, Yuting Luo, Jing Hong, Huacheng He, Lu Dai, Hong Zhu, Jiang Wu

**Affiliations:** ^1^ Department of Endocrinology, The First Affiliated Hospital of Wenzhou Medical University, Wenzhou, Zhejiang, China; ^2^ Key Laboratory of Biotechnology and Pharmaceutical Engineering, School of Pharmaceutical Sciences, Wenzhou Medical University, Wenzhou, Zhejiang, China; ^3^ College of Chemistry and Materials Engineering, Wenzhou University, Wenzhou, Zhejiang, China; ^4^ The Fourth Outpatient Department, The Affiliated Stomatological Hospital of Nanjing Medical University, Nanjing, Jiangsu, China

**Keywords:** biomaterials, lower extremity arterial disease, therapeutic angiogenesis, critical limb ischemia, peripheral arterial disease

## Abstract

Lower extremity arterial disease (LEAD) is a major vascular complication of diabetes. Vascular endothelial cells dysfunction can exacerbate local ischemia, leading to a significant increase in amputation, disability, and even mortality in patients with diabetes combined with LEAD. Therefore, it is of great clinical importance to explore proper and effective treatments. Conventional treatments of diabetic LEAD include lifestyle management, medication, open surgery, endovascular treatment, and amputation. As interdisciplinary research emerges, regenerative medicine strategies have provided new insights to treat chronic limb threatening ischemia (CLTI). Therapeutic angiogenesis strategies, such as delivering growth factors, stem cells, drugs to ischemic tissues, have also been proposed to treat LEAD by fundamentally stimulating multidimensional vascular regeneration. Recent years have seen the rapid growth of tissue engineering technology; tissue-engineered biomaterials have been used to study the treatment of LEAD, such as encapsulation of growth factors and drugs in hydrogel to facilitate the restoration of blood perfusion in ischemic tissues of animals. The primary purpose of this review is to introduce treatments and novel biomaterials development in LEAD. Firstly, the pathogenesis of LEAD is briefly described. Secondly, conventional therapies and therapeutic angiogenesis strategies of LEAD are discussed. Finally, recent research advances and future perspectives on biomaterials in LEAD are proposed.

## Graphical Abstract

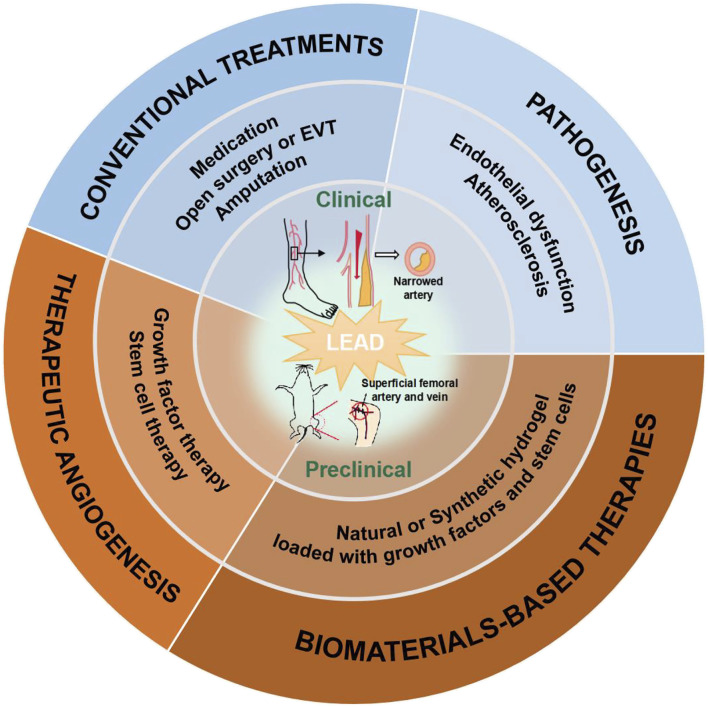

## 1 Introduction

**FIGURE 1:**
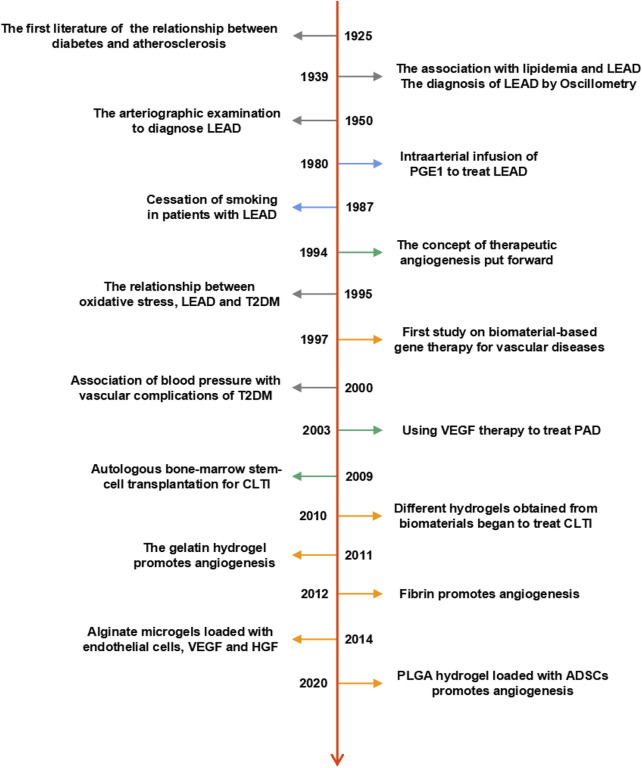
Timeline of preclinical and clinical research on LEAD. The dates listed indicate essential milestones in the history of LEAD.

Lower extremity arterial disease (LEAD) is a critical component of peripheral arterial disease (PAD), which defined as a vascular disease caused by occlusion of lower extremity arteries. It is widely recognized that LEAD is associated with type 2 diabetes mellitus (T2DM) (Fowkes et al., 2017). LEAD is an essential factor contributing to the increased mortality in T2DM patients (Nativel et al., 2018). Over the past century, preclinical and clinical research of LEAD have been intensively studied. Here, Figure 1 presented the time line of importance matter of LEAD. Since 1925, the risk factors associated with T2DM and LEAD have been gradually revealed, such as hyperlipidemia (Barker, 1939), hypertension (Adler et al., 2000) and oxidative stress (Belch et al., 1995). Meanwhile, different diagnosis methods (Naide, 1939; Campbell and Smith, 1950) and conventional treatments (Sethi et al., 1980; Jonason and Bergstrom, 1987) of LEAD have also emerged. For instance, there are several treatments commonly used in clinical practice, such as lifestyle management, medication, open surgery, and endovascular revascularization (Teraa et al., 2016). These treatments can increase blood perfusion and restore microcirculation in the ischemic limbs. Unfortunately, these may be ineffective in some patients.

In 1994, the concept of therapeutic angiogenesis was put forward (Takeshita et al., 1994), and since then, researchers have started to use growth factors (Rajagopalan et al., 2003) or stem cells (Amann et al., 2009) to treat LEAD. With the continuous progress of medicine, therapeutic angiogenesis arises at the historical moment with aims to promote revascularization by changing the pathological conditions around ischemic tissues (Xing et al., 2021a). At present, therapeutic angiogenesis mainly includes growth factor therapy, stem cell therapy and gene therapy (Veith et al., 2019). However, since most studies remain in the laboratory stage, further clinical tests are needed to verify the efficiency of therapeutic angiogenesis in people who suffer from LEAD.

Biomaterial-based gene therapy for vascular diseases was introduced in 1997 (Schwartz and Moawad, 1997); from then on, researchers have turned their attention to the field of biomaterials (Delcroix et al., 2010; Moon et al., 2011; Agudelo et al., 2012; Kim et al., 2014; Hong et al., 2020). Biomaterials become suitable carriers for encapsulating and delivering biologically active substances or drugs, which provide new therapeutic ideas for promoting angiogenesis in diabetes patients with LEAD. Among different kinds of biomaterials, hydrogel has been extensively studied. Researchers have used natural or synthetic materials to create various hydrogels with diverse biological properties, of which injectable hydrogel has proved to improve blood perfusion in animals with lower limb ischemia. Given many advantages, such as biocompatibility, biodegradability and non-toxicity, hydrogel and other biomaterials, for example, scaffold, nanoparticle and spheroid, are hopefully being used in clinical research to increase the therapeutic efficiency of diabetic LEAD.

## 2 Overview of diabetic LEAD

The 10th edition of the IDF Diabetes Atlas indicates that over 500 million people worldwide are affected by T2DM (Sun et al., 2022), and it is reported that the number of people who suffer from LEAD are over 200 million globally (Fowkes et al., 2013). Compared to non-diabetic people, patients with both T2DM and LEAD show a worse prognosis, for example, the increased risks of CLTI, major adverse limb events (MALEs) and major adverse cardiac events (MACEs).

Although the pathogenesis of diabetic LEAD is still not fully clarified, it is believed that the key factor contributing to LEAD is atherosclerosis. The hallmark of atherosclerosis is endothelial dysfunction, which is caused by multiple interrelated risk factors, such as the overproduction of advanced glycation end products (AGEs), excessive oxidative stress and inflammation (Soyoye et al., 2021). Besides, the migration of low-density lipoprotein cholesterol (LDL-C) to the intima is a precursor of atherosclerosis (Suzuki et al., 2001; Taniwaki et al., 2001; Yang et al., 2017). Endothelial dysfunction and hyperglycemia are associated with abnormal clotting. Thus, abnormal platelet activation and aggregation are important factors in the development of atherosclerosis (American Diabetes, 2003).

Based on the pathological factors identified so far, researchers have further explored advanced treatments for LEAD by establishing animal models of limb ischemia. Ligation or resection of superficial femoral artery and vein are one of the most commonly used surgical methods (Masaki et al., 2002; Tan et al., 2007; Wang et al., 2020a). However, as the ligation and resection result in a state of acute ischemia, this differs from the vascular pathology of the lower extremity in clinical diabetic patients, whose vascular disease developed after a long period of ischemia. Luckily, this animal model is suitable for studying new strategies to treat LEAD due to the severe degree of ischemia and long postoperative blood flow recovery time, which simulates the vascular state of CLTI patients.

## 3 Conventional treatments of diabetic LEAD

Conventional treatments of diabetic LEAD include: 1) medication; 2) open surgery or endovascular treatment; 3) amputation; and 4) lifestyle management. Firstly, a good lifestyle is a needed for all other treatments. Secondly, patients are advised to take their medication as prescribed by their doctor. In addition, open surgery and endovascular treatment are indicated in patients for whom medication has failed or been ineffective. Finally, people with CLTI may experience an amputation. Here, we will describe each of these four treatments.

### 3.1 Medication

The drug treatments are also commonly used in clinical practice to slow down the progression of LEAD, or prevent acute cardiovascular or limb events.

Firstly, glycemic control is a primary treatment for patients with diabetic LEAD. The research revealed that combining glucagon-like peptide-1receptor agonists (GLP-1RAs) (e.g., liraglutide) with standard treatment can decrease amputation in T2DM people who suffer from diabetic foot ulcers (DFU) (Dhatariya et al., 2018). However, the relationship between sodium-glucose co-transporter 2 inhibitors (SGLT2is) and lower limb amputation remains unclear; the Canagliflozin Cardiovascular Assessment Study (CANVAS) reported that canagliflozin increased amputation rates (Minami et al., 2021). A meta-analysis showed no increased risk for SGLT2is compared to placebo (Heyward et al., 2020). A synthesis of the current studies leads to the conclusion that SGLT2is does not increase the risk of amputation and that GLP-1RA reduces this risk.

Secondly, the incidence of LEAD in DM patients is greatly associated with blood pressure (Mohammedi et al., 2016). The ACC and AHA guidelines suggest using angiotensin-converting enzyme inhibitors (ACEIs) or angiotensin receptor antagonists (ARBs) for patients with PAD (Gerhard-Herman et al., 2017).

Thirdly, high LDL-C is related to atherosclerosis, which leads to LEAD (Gebauer and Reinecke, 2018). There are large amounts of evidence prove the benefits of statins for LEAD (Gerhard-Herman et al., 2017). In addition, apolipoprotein B (apoB) and non-high-density lipoprotein cholesterol (non-HDL-C) have been shown to predict CVD, even at low levels of LDL-C (McQueen et al., 2008). A recent study suggests that increased non-HDL and apoB are better predictors of arterial stiffness than LDL-C (Jia et al., 2022).

Finally, patients with ischemia symptoms should be treated with anti-platelet or anticoagulation therapy. The AHA/ACC guidelines suggest using aspirin or clopidogrel alone to reduce vascular risk and mortality (Gerhard-Herman et al., 2017). As an anticoagulant, rivaroxaban has proven benefit in PAD. In the VOYAGER PAD trial, rivaroxaban 2.5 mg twice daily plus low-dose aspirin significantly reduced acute limb, brain and cardiac ischemic events compared with aspirin alone (Bonaca et al., 2020).

### 3.2 Open surgery and endovascular treatment

Lower extremity open surgery and endovascular treatment (EVT) have been used to restore blood perfusion in patients with CLTI (Farber and Eberhardt, 2016). EVT has the advantage of a simpler and less time-consuming procedure, as well as a faster recovery and lower recurrence rate (Papavassiliou et al., 2003; Adam et al., 2005; Slovut and Lipsitz, 2012). However, neither open surgery nor EVT can prevent restenosis (Biscetti et al., 2021). LEAD patients often accompany with occlusion of the distal arteries in the calf and foot, there will be a lack of suitable arterial inflow as well as an adequate vascular bed, leading to the failure of surgical treatment (Pan et al., 2019).

### 3.3 Amputation

Amputation is the ultimate clinical treatment for patients with CLTI (Norgren et al., 2007). It includes minor and major amputation. Minor amputation barely affects the patient’s motor function, whereas major amputation is usually performed above the ankle joint, reducing the independent motor ability after surgery (Nazri et al., 2019). It will seriously affect the quality of life of patients, so clinicians should intervene in the early stages of LEAD to reduce the occurrence of amputation.

### 3.4 Lifestyle management

As we known, lifestyle changes are an efficient way in treating abnormal glucose tolerance. However, most patients are not very compliant and have difficulty adhering to good lifestyle habits. We recommend all pre-diabetics and diabetics to initiate lifestyle management which will reduce the incidence of diabetic complications. Firstly, smoking ranks first in all risk factors for PAD. Stopping smoking can slow the procession of limb ischemia and reduce amputation, even mortality in patients (Armstrong et al., 2014). Secondly, the LEAD patient need physical exercise along with glycemic control, this benefit weight loss, lowering blood pressure and improving insulin sensitivity (Abrignani, 2018; Lee et al., 2018). It also helps patients alleviate ischemic symptoms and decrease the incidence of cardiovascular disease (CVD) (Lane et al., 2017).

Diet is an important way to both prevention and treatment of diseases, including DM (Martin-Pelaez et al., 2020). Studies have demonstrated that the Geriatric Nutrition Risk Index (GNRI) and Controlled Nutritional Status (CONUT) scores are predictors of prognosis for patients with LEAD. Therefore, improving malnutrition is a new therapeutic target in elder population (Yokoyama et al., 2018; Jhang et al., 2020).

## 4 Therapeutic angiogenesis

Although conventional treatments of diabetic LEAD are commonly used in the clinical stage, the methods mentioned above still have certain limitations, such as the inability to allow revascularization of the ischemic limb itself (Xing et al., 2021a). Moreover, the overall prognosis is less than ideal, and the mortality after amputation remains high. To fill these gaps, researchers have explored “therapeutic angiogenesis” since the 1990s. It mainly includes protein therapies, gene and nucleic acid-based therapies, drug and drug-like compounds therapies and stem cell-based therapies (Veith et al., 2019). Therapeutic angiogenesis strategies are extensively studied in animal experiments, and the safety of growth factor and stem cell-based biologic therapies has also been confirmed in randomized clinical trials.

### 4.1 The conception of therapeutic angiogenesis

**FIGURE 2:**
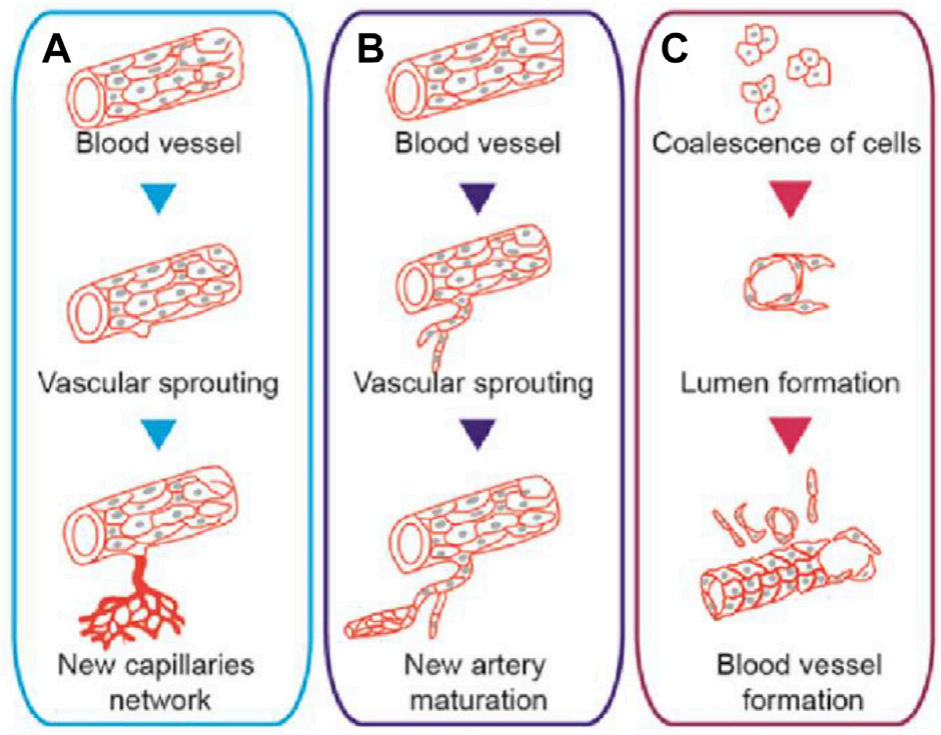
Schematic diagram of different types of therapeutic angiogenesis. **(A)** Angiogenesis: revascularization formed through capillary sprouting based on the intrinsic vessels. **(B)** Arteriogenesis: collateral vessel formation and artery maturation through capillary sprouting. **(C)** Vasculogenesis: blood vessels formed through the accumulation of EPCs. Figure adapted with permission from ref. (Xing et al., 2021a).

Therapeutic angiogenesis was firstly proposed in an experiment in which vascular endothelial growth factor (VEGF) was injected into the ischemic hind limb of rabbits to demonstrate revascularization (Takeshita et al., 1994). Therapeutic angiogenesis can be divided into three types (Figure 2) (Xing et al., 2021a): 1) Angiogenesis: a new capillary network is sprouted based on the inherent blood vessels. This process includes the activation of VEGF, hypoxia-inducible factor-1α (HIF-1α) and erythropoietin (EPO). These cytokines act on endothelial cells and promote angiogenesis; 2) Arteriogenesis: including collateral vessel formation and artery maturation (Chalothorn et al., 2007). When the microenvironment changes, the expression of tumor necrosis factor (TNF) and fibroblast growth factor (FGF) increases, which further induces arteriogenesis (de Muinck and Simons, 2004); 3) Vasculogenesis: new blood vessels form *in situ*. Firstly, endothelial progenitor cells (EPCs) gather with each other, then fuse with the surrounding cells, gradually form the vascular lumen structure, and finally become the mature blood vessel (Kamei et al., 2006; Xing et al., 2020). With more in-depth studies of therapeutic angiogenesis, researchers found that this novel treatment strategy can help the body form endogenous revascularization and restore blood perfusion in ischemic tissue. Therefore, therapeutic angiogenesis is expected to become a new scheme to treat limb ischemia in patients with diabetic LEAD.

### 4.2 Growth factor therapy

**FIGURE 3:**
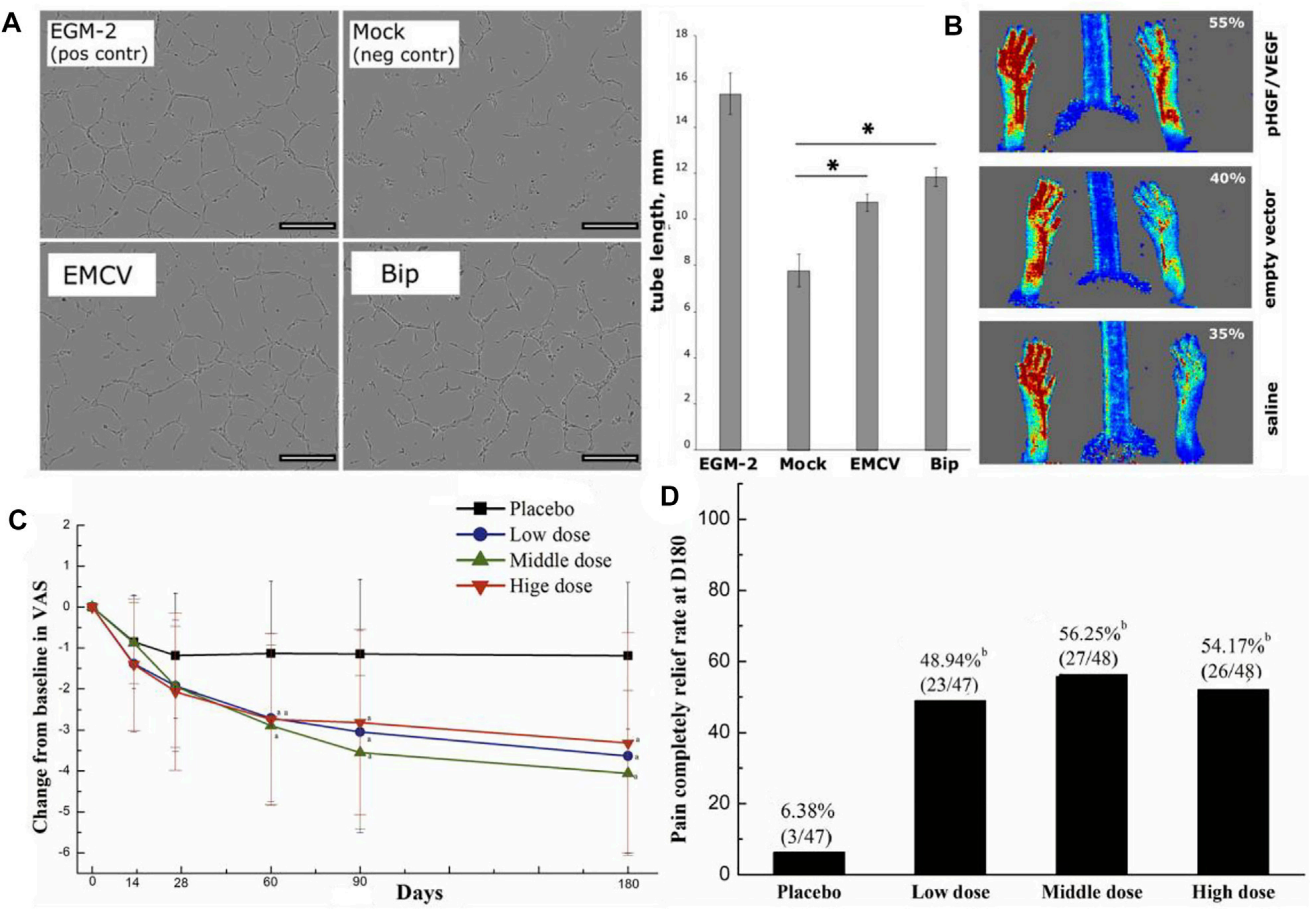
Schematic diagrams of growth factor therapy to improve angiogenesis. **(A)** Tube formation assay of HUVECs after HGF/VEGF165 plasmid treatment. Scale bar = 300 μm. **(B)** Laser doppler perfusion imaging (LDPI) images of ischemic hind limbs and analytical diagrams of the perfusion rate. **(C)** The relief of rest pain after HGF plasmid treatment. **(D)** Rate of completely pain relief at the end of HGF plasmid treatment. **(A,B)** are reprinted with permission from ref. (Slobodkina et al., 2020) and **(C,D)** are reprinted with permission from ref. (Gu et al., 2019).

At present, many studies have been conducted on growth factors such as FGF, VEGF, HGF, PDGF, and EGF to induce angiogenesis. Interestingly, in the experimental animal stage, exogenous growth factors showed an excellent function of stimulating ischemic collateral circulation; however, the effect was slightly inadequate in clinical trials. Makarevich et al. (2012) demonstrated that intramuscular injection of a mixture of plasmids that contain VEGF 165 and HGF could restore the blood flow in ischemic skeletal muscle. Upon this, they developed a novel bicistronic plasmid expressing both VEGF165 and HGF. The results showed that HGF/VEGF165 plasmid could induce angiogenesis in human umbilical vein endothelial cells (HUVECs) and improve blood perfusion in a hind limb ischemia mouse model (Figures 3A,B) (Slobodkina et al., 2020). Regarding the effectiveness of growth factor therapy in clinical trials, its therapeutic effect on patients with LEAD is less significant. Gu et al. studied the efficiency of HGF plasmid NL003 for the treatment of limb ischemia. They found those 6 months after intramuscular injection of NL003, patients experienced relief of ischemic symptoms and had a higher rate of ulcer healing. However, no statistical differences were found in transcutaneous oxygen pressure (TcPO_2_), ankle-brachial index (ABI), or toe-brachial index (TBI) in both control and experimental groups (Figures 3C,D) (Gu et al., 2019). It shows that the clinical value of growth factors in LEAD patients still needs a large number of in-depth studies to verify their effectiveness.

### 4.3 Stem cell therapy

**FIGURE 4:**
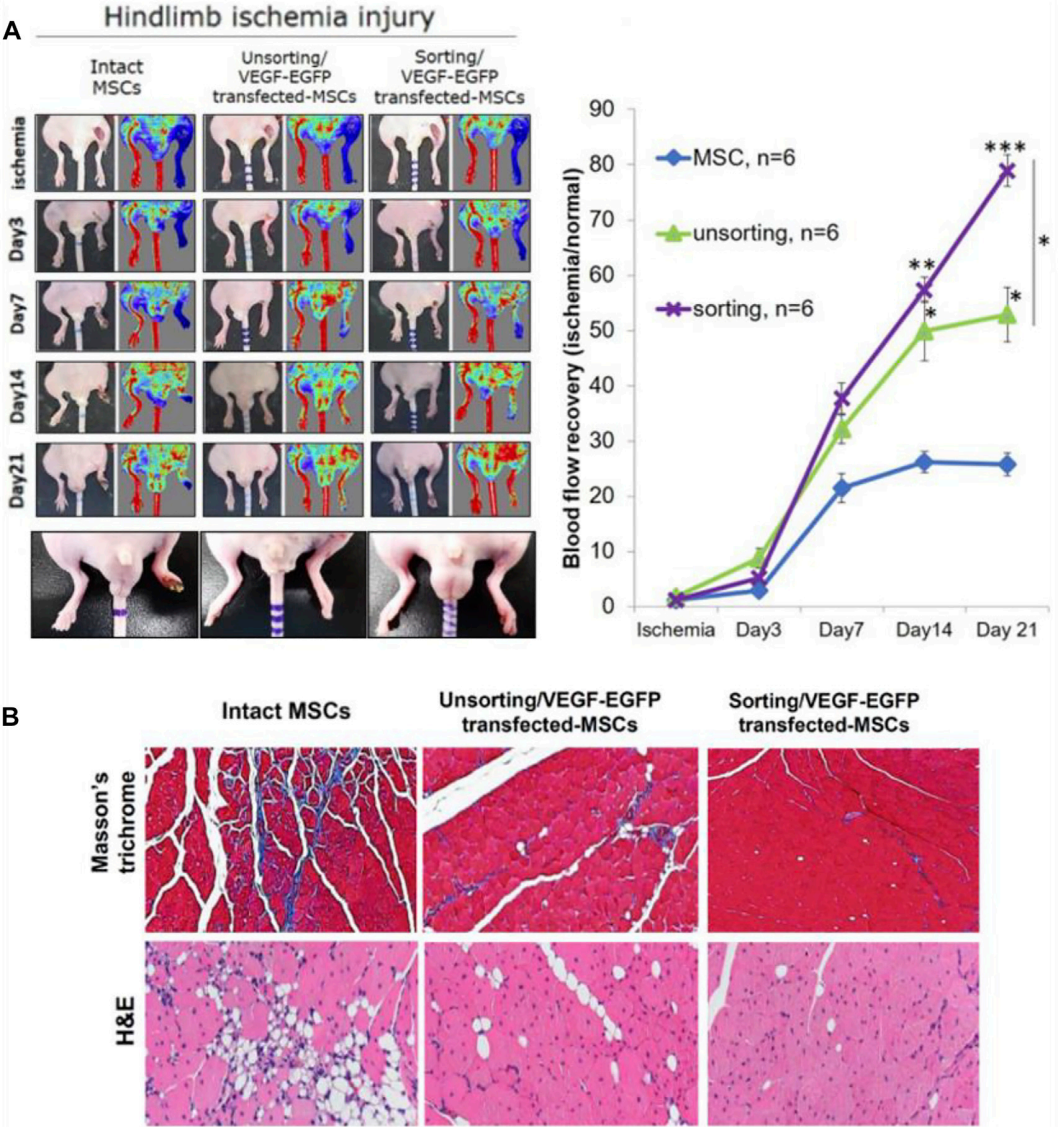
Schematic diagrams of MSCs transplantation to treat hind limbs ischemia in a mouse model. **(A)** LDPI images of ischemic hind limbs and analytical graphs of the perfusion recovery rate. **(B)** Representative pictures of Masson’s trichrome staining showing fibrosis and H&E staining showing necrosis. Figure adapted with permission from ref. (Park et al., 2019).

Stem cell therapy can be used for patients who cannot undergo open surgery or EVT. Many types of stem cells are already being explored, like EPCs, bone marrow mononuclear cells (BMMNCs), peripheral blood mononuclear cells (PBMNCs) and mesenchymal stem cells (MSCs), etc. At present, numerous researches have proved the effectiveness, such as improving blood perfusion, relieving rest pain and increasing ABI and TcPO_2_ (Jonsson et al., 2012; Moazzami et al., 2014; Rigato et al., 2017). In addition to conducting studies about stem cell transplants, researchers have also combined growth factors with stem cells to explore their effects on angiogenesis. Park et al. (2019) used VEGF and enhanced green fluorescent protein (EGFP) to transfect MSCs, and then transplanted the MSCs into the ischemic hind limbs to observe their effect of revascularization. They found that this MSCs-based transplantation therapy could not only enhance the expression of VEGF, but also improve blood perfusion and induce angiogenesis (Figure 4).

Similar to growth factor therapy, the results of stem cell therapy in clinical trials are not as favorable as in animal experiments, and the results vary from one researcher to another. Ai et al. (2016) and Rigato et al. (2017) found that cell-based transplantation may reduce amputation and increase the survival rate of non-amputation. Xie et al. (2018) and Gao et al. (2019) found that although the total amputation rate decreased after stem cell therapy, it did not improve the preservation of major limbs. In addition, a large-scale randomized controlled trial showed that the total amputation rate, mortality and non-amputation survival rate in the treatment group might not be significantly improved after stem cell transplantation (Peeters Weem et al., 2015; Liew et al., 2016; Pan et al., 2018). It remains unclear whether stem cell therapy can actually benefit patients with diabetic LEAD, we sincerely hope that its efficacy can be verified and therefore we summarize some of the current studies on stem cell therapy in Table 1.

**TABLE 1:** Clinical trials based on cell therapy for LEAD patients.

Author	Year	Cell type	Study population	Delivery	Outcome	Mean follow-up	Refs.
Lara et al.	2010	EPCs	28	Intramuscular injection	Increased ABI, decreased pain, improved tissue perfusion and obtained high amputation-free rates	14 months	Lara-Hernandez et al. (2010)
Benoit et al.	2011	BMSCs	48	Intramuscular injection	Decreased amputation and improved ulcer healing	6 months	Benoit et al. (2011)
Ozturk et al.	2012	PBMNCs	40	Intramuscular injection	Increased ABI, TcPO2, and 6-min walking distance, decreased pain score and limb ulcers	12 months	Ozturk et al. (2012)
Lasala et al.	2012	MSCs and EPCs	26	Intramuscular injection	Improved walking time and ABI, increased perfusion	4 months	Lasala et al. (2012)
Szabo et al.	2013	PBSCs	20	Intramuscular injection	Increased ulcer healing and TcPO2	24 months	Szabo et al. (2013)
Gupta et al.	2013	BMMSCs	20	Intramuscular injection	Increased ABI, decreased amputation, and improved ulcer healing	6 months	Gupta et al. (2013)
Skóra et al.	2015	BMMNCs	32	Intramuscular injection	Increased ABI, developed collateral vessels, and decreased amputation	3 months	Skora et al. (2015)

Based on current research reports, stem cell-based therapies may have better efficacy than other therapeutic angiogenesis strategies (Ho et al., 2022), as cell therapy has been shown to induce therapeutic angiogenesis in both animal models and human experiments (Van Raemdonck et al., 2015; Novakova et al., 2016). Compared to other stem cells, induced pluripotent stem cells (iPSC) and their derivative cells may have enhanced therapeutic potential. It reported that human iPSC-derived smooth muscle cells (hiPSC-SMC) can mediate angiogenesis, thereby improving hind limb ischemia. Stem cell therapy using iPSC-derived cells may represent a novel therapeutic angiogenesis strategy for the treatment of LEAD (Gao et al., 2021; Park et al., 2021).

The therapeutic angiogenesis strategy was carried out as early as 1990s and it is currently under great investigation. Although promising preclinical studies in animal models, cell-based therapies in LEAD patients still show limitations. It has been demonstrated that ion channels differ between humans and animal models and that the disease state in humans cannot be fully replicated in animal models (Tanner and Beeton, 2018) . Most previous investigations used autologous cells, which damaged by chronic hyperglycemia, and have a low survival rate in the ischemic environment (Qadura et al., 2018). However, we can initially screen some effective drugs through animal studies, and then verify their safety and effectiveness through clinical trials. In the future, multicenter, larger-scale clinical trials are needed to verify the effectiveness of therapeutic angiogenesis in clinical LEAD patients.

## 5 Application of biomaterials in the treatment of diabetic LEAD

Researchers have verified the effectiveness of therapeutic angiogenesis in the experimental animal stage, but when it turns to clinical trials, this therapeutic strategy remains to be further explored. At present, most studies transport growth factors, stem cells or other bioactive molecules to ischemic tissue in order to restore blood perfusion. However, how to maintain the activity of these substances and how to improve the accuracy of delivery still requires more in-depth research. Due to the excellent biocompatibility, biodegradability and non-toxicity, biomaterials can be used as a carrier of various bioactive molecules to treat LEAD. As reported, the vascular scaffold has been used in patients with PAD, and no amputations or deaths have been observed over a 2-years clinical trial (Lammer et al., 2016). Therefore, biomaterials are of great clinical importance in the treatment of LEAD.

### 5.1 Biomaterials overview

**FIGURE 5:**
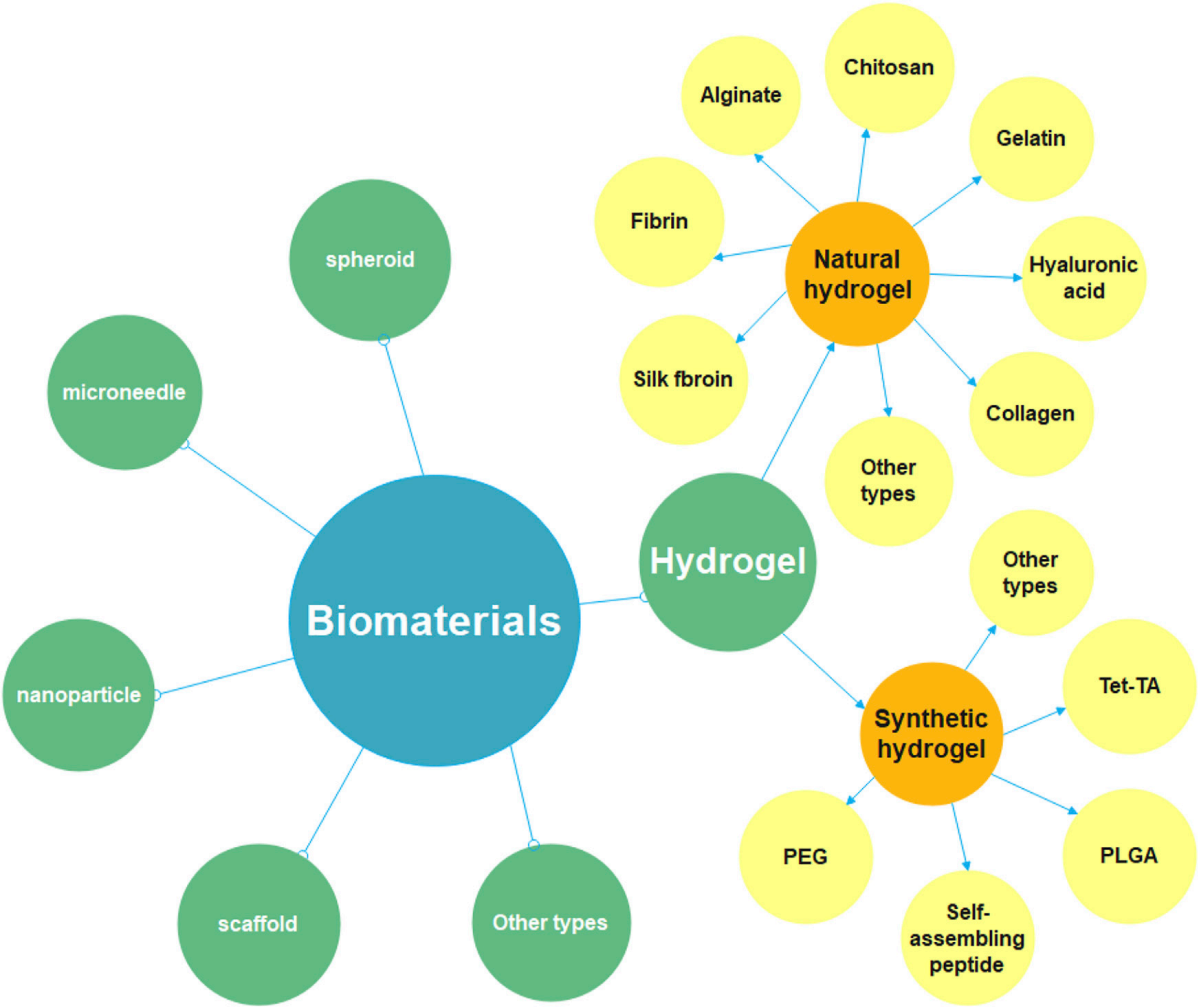
Diagram of the classification of biomaterials. Biomaterials include hydrogel, spheroid, microneedle, nanoparticle, scaffold and so on, this review focuses on hydrogel-related research. It can be divided into natural hydrogel and synthetic hydrogel. The natural materials for preparing hydrogels include chitosan, gelatin, fibrin, alginate, collagen, hyaluronic acid, silk fibroin, etc. The synthetic materials for preparing hydrogels include polyethylene glycol (PEG), poly (lactic-co-glycolic Acid) (PLGA), self-assembling peptide (SAP), tetronic-tyramine (Tet-TA), etc.

Biomaterials are one of the significant components of tissue engineering, acting as an extracellular matrix (ECM) and promoting cell migration, tube formation, tissue maturation and wound healing (Zhang et al., 2013; Lee et al., 2014). There are numerous ways to classify biomaterials; for example, according to the origins, biomaterials can be classified as natural or synthetic materials (Troy et al., 2021). Researchers use these biomaterials to prepare tissue engineering materials with different structural forms and diverse functions, such as hydrogels, microneedles, nanoparticles and scaffolds, etc. (Figure 5). Taking hydrogel as an example, on the one hand, it has good biological properties and mechanical properties; on the other hand, the preparation is more straightforward compared with other biomaterials. Therefore, in biomaterial-based therapeutic angiogenesis strategies, hydrogels have become promising candidates (Buwalda et al., 2017). Here, we will mainly focus on some of the explorations that have been completed in the field of hydrogels and list some studies in the treatment of LEAD based on other biomaterials.

### 5.2 Hydrogel overview

Hydrogel is a kind of hydrophilic polymer material, which is extensively used in the medical stage because of its excellent water absorption, biocompatibility, and ion transferability (Xing et al., 2021a). Due to the conventional treatments of LEAD and therapeutic angiogenesis strategies having certain limitations and side effects, researchers have made an in-depth exploration of therapeutic angiogenesis based on hydrogels. They have designed various studies to treat LEAD by encapsulating various angiogenic factors or stem cells in hydrogels and then transporting them to ischemic tissues effectively. According to the different sources of materials for preparing hydrogel, it can be divided into natural hydrogel and synthetic hydrogel.

#### 5.2.1 Natural hydrogel

**FIGURE 6:**
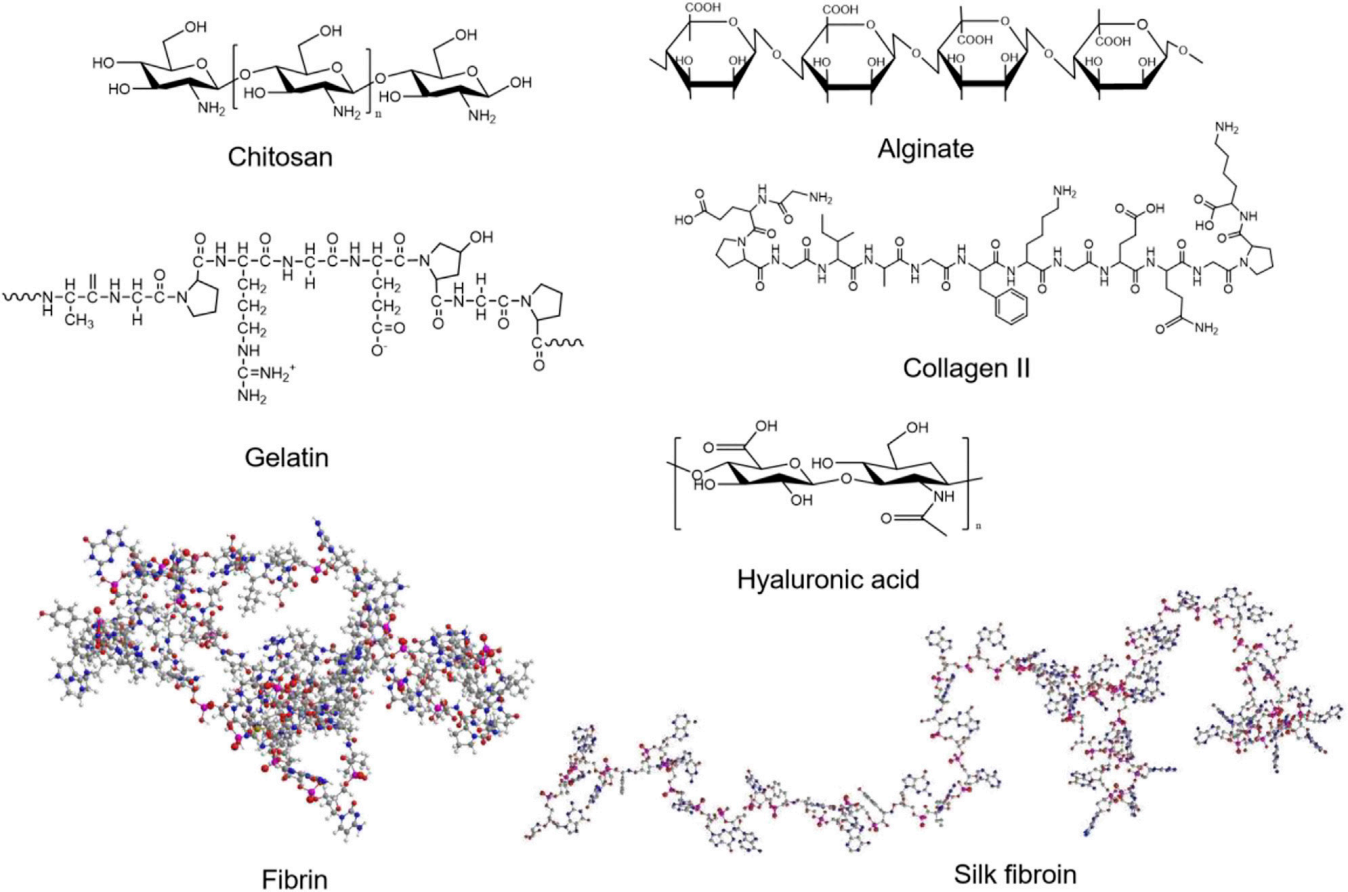
The structure of natural materials for preparing hydrogels.

**FIGURE 7:**
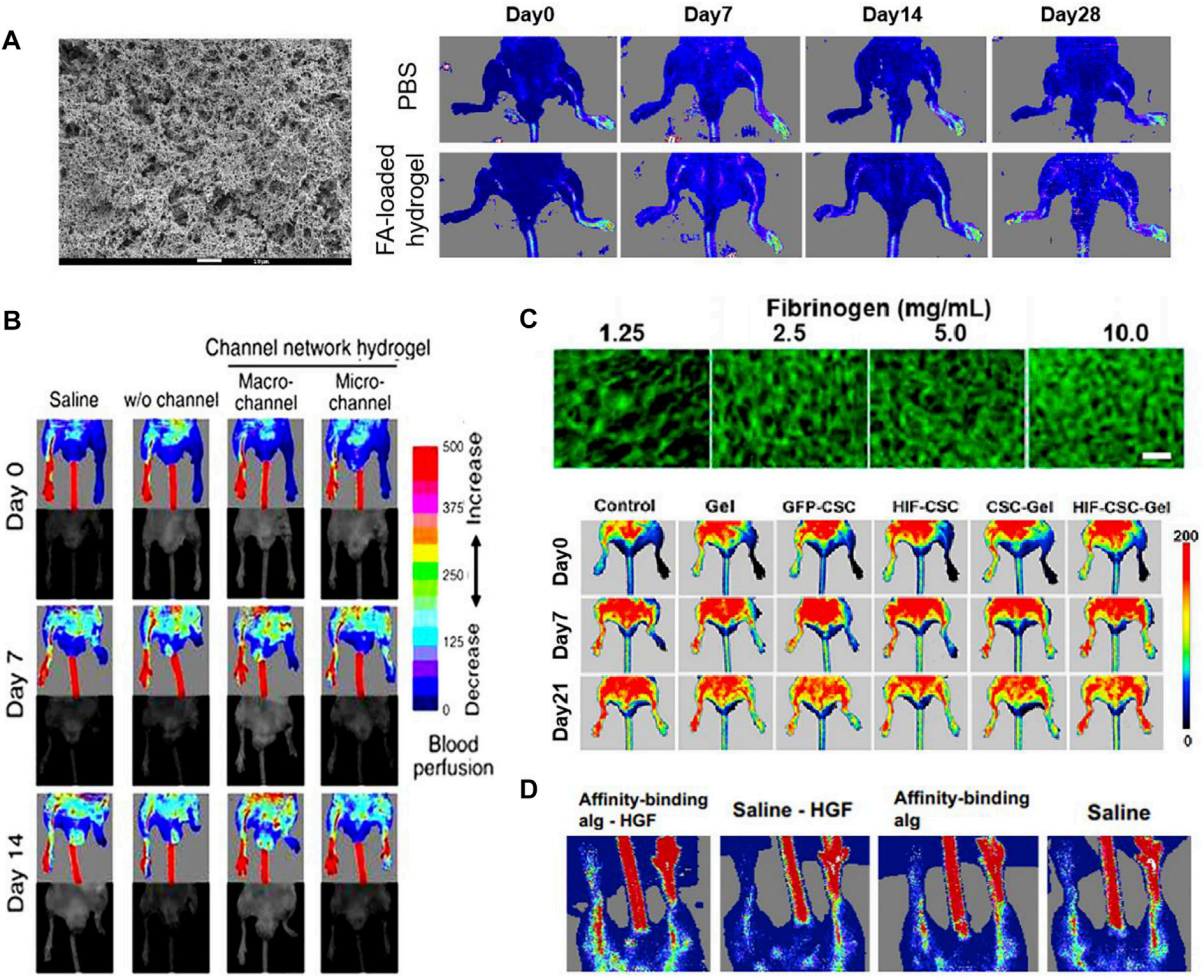
Schematic diagrams of several natural hydrogels to improve angiogenesis. **(A)** The SEM image of the FA-chitosan hydrogel and the result of animal study to promote angiogenesis. Scale bar = 10 μm. **(B)** LDPI images in a hind limb ischemia mouse model. **(C)** Confocal microscopy images of a micro-architecture of injectable fibrin hydrogel with different concentrations of fibrinogen solution and the representative LDPI images. Scale bar = 50 μm. **(D)** LDPI images proved the angiogenesis effect of the HGF-alginate hydrogel. **(A)** is reprinted with permission from ref. (Wang et al., 2020b). **(B)** is reprinted with permission from ref. (Lee et al., 2020). **(C)** is reprinted with permission from ref. (Pei et al., 2020). **(D)** is reprinted with permission from ref. (Ruvinov et al., 2010).

The natural materials for preparing hydrogels include chitosan (CS), gelatin, fibrin, alginate, collagen, hyaluronic acid (HA), silk fibroin (SF), etc. (Figure 6). Chitosan has been broadly used in biomedical fields for its biocompatibility, degradability and antibacterial (Jayakumar et al., 2010; Kalantari et al., 2019). Recently, Wang et al. (2020b) synthesized a temperature-responsive chitosan hydrogel encapsulated with ferulic acid (FA). The hydrogel is capable of the stable release of FA, which has the effects of both antioxidant and pro-angiogenic. They validated the efficacy of the FA-chitosan hydrogel in a hind limb ischemia mouse model and demonstrated its remarkable ability to promote blood perfusion and angiogenesis (Figure 7A). Gelatin has good temperature responsiveness; when the temperature is below 40°C, it forms into a transparent gel solution (Chu and Wang, 2012). Lee et al. (2020) prepared a microchannel network hydrogel with gelatin solution and poly (N-isopropyl acrylamide) (PNIPAM) fibers. The *in vivo* results proved that this hydrogel could increase blood perfusion (Figure 7B). Fibrin has a large number of binding sites to growth factors, which allows angiogenesis (Loureiro et al., 2019). Recently, Pei et al. (2020) prepared a fibrin hydrogel encapsulating cardiac stem cells (CSCs) and transfected HIF-1α into CSCs. The *in vivo* results of animal experiments showed that the hydrogel has a solid ability to promote angiogenesis and restore the blood perfusion of ischemic limbs (Figure 7C). Alginate is a kind of natural anionic polymer, which is widely used in the preparation of hydrogel due to its easy gelation. Ruvinov et al. (2010) prepared an alginate hydrogel encapsulated with HGF, which released HGF in a rapid and controlled manner. Then, they established a mouse model to verify the effect of the HGF-hydrogel. The *in vivo* results proved that the HGF-hydrogel could improve blood perfusion by improving angiogenesis through its pro-angiogenesis effect (Figure 7D). Since many research used natural hydrogels for LEAD, we summarized some of studies in Table 2.

**TABLE 2:** Preclinical studies of natural hydrogels for LEAD.

Materials	Cargo	Animals	Injury model	Blood perfusion	Revascularization	Others	Refs.
Chitosan	VEGF, ECs, CD31^+^ cells	Mice	Femoral artery ligation	Increased	Increased capillary density	Increased mRNA level of angiogenic factors	Lee et al. (2015)
Chitosan‐HP	FGF-2	Rats	Femoral artery ligation	Increased	Increased capillary density	Enhanced early vascular maturation	Nillesen et al. (2007)
Alginate	HGF	Mice	Femoral artery excised	Increased	Increased arteriole density	Not mentioned	Ruvinov et al. (2010)
Alginate	IGF + VEGF	Rabbits	Lateral circumflex artery and femoral artery ligation	Increased	Increased capillary density	Increased muscle fibers size	Anderson et al. (2017)
Alginate	CD4^+^ T cells	CD4 KO mice	Femoral artery and vein ligation	Increased	Increased blood vessel density	Enhanced myogenesis	Kwee et al. (2018)
Gelatin	G-CSF, FGF-2	Mice	Femoral artery excised	Increased	Increased capillary density	Promoted mature vessel formation	Layman et al. (2009)
Gelatin	FGFs, plasma derived GFs mixture	Mice	Iliac and femoral artery and vein excised	Increased	Increased blood vessel density	Not mentioned	Matsui and Tabata, (2012)
Fibrin	HIF-1α overexpressed CSCs	Mice	Femoral artery excised	Increased	Not mentioned	Attenuated tissue degeneration and fibrosis	Pei et al. (2020)
Fibrin	Recombinant VEGF164	Mice	Femoral artery excised	Increased	Increased the mature capillaries	Improved wound-healing	Sacchi et al. (2014)
Fibrin	FGF2, HO-1 gene	Nude mice	Femoral artery excised	Not mentioned	Not mentioned	Reduced tissue apoptosis and necrosis, enhanced angiogenic paracrine factor expression	Bhang et al. (2009)
Collagen‐HP	FGF-2	Rabbits	Femoral artery excised	Not mentioned	Increased capillary density	Increased oxygen perfusion	Zhou et al. (2012)
Catechol modified HA	ADSCs	Nude mice	Femoral artery excised	Increased	Increased capillary and arteriole density	Attenuated tissue degeneration and fibrosis	Park et al. (2016)

#### 5.2.2 Synthetic hydrogel

**FIGURE 8:**
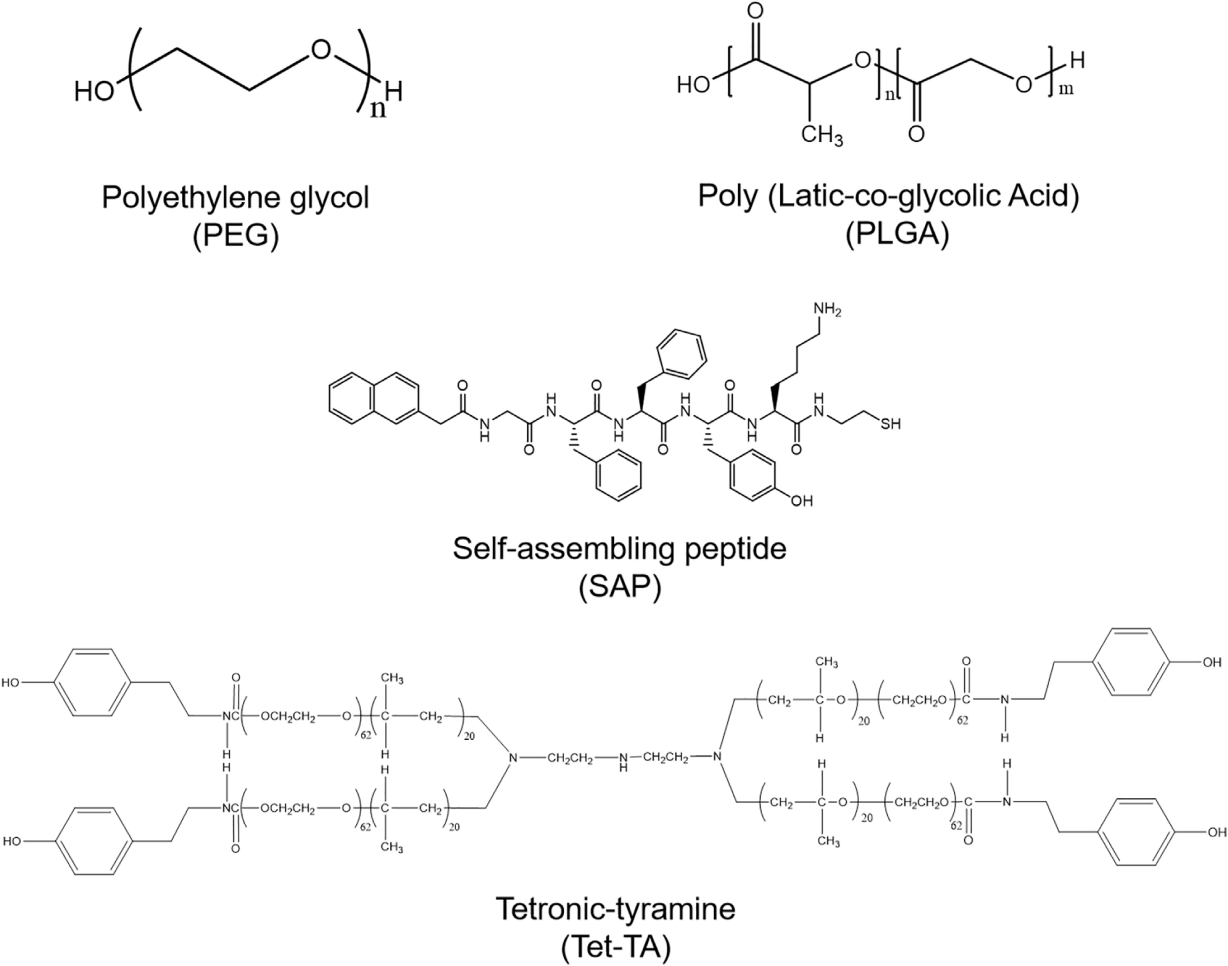
The structures of synthetic materials for preparing hydrogels.

**FIGURE 9:**
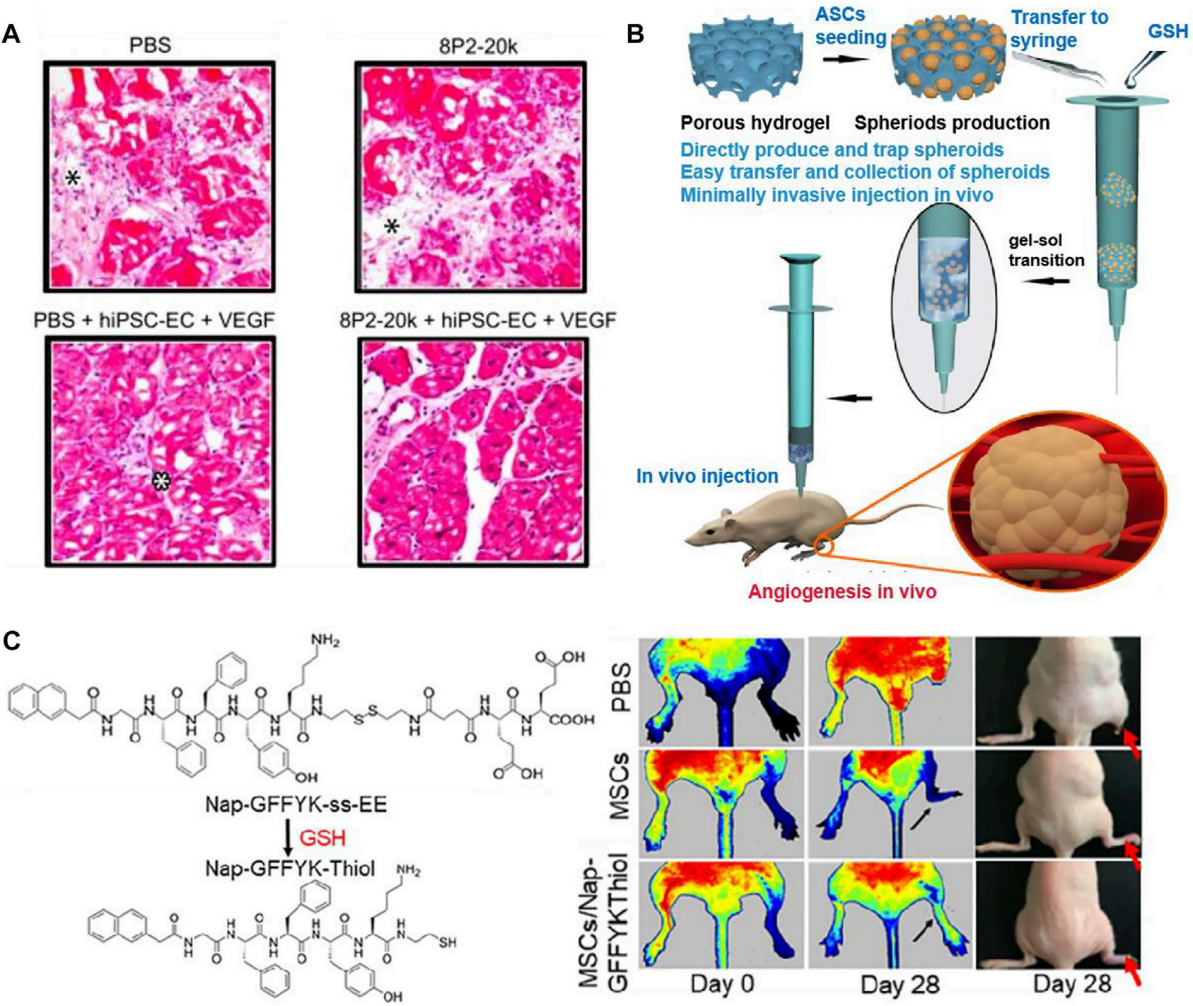
Schematic diagrams of several synthetic hydrogels to improve angiogenesis. **(A)** Representative images of H&E staining showed the effect of PEG hydrogel, which co-deliver hiPSC-ECs and VEGF. Only the protein-PEG co-deliver system group showed no significant inflammation. Black asterisk indicates the expansion of connective tissue and white asterisk indicates inflammatory cells. **(B)** General diagram of the preparation of PLGA-Cys-mPEG porous hydrogel and the *in vivo* result of angiogenesis. **(C)** Chemical structures of Nap-GFFYK-Thiol hydrogel and LDPI images of blood perfusion. **(A)** is reprinted with permission from ref. (Mulyasasmita et al., 2014). **(B)** is reprinted with permission from ref. (Hong et al., 2020). **(C)** is reprinted with permission from ref. (Huang et al., 2019a).

The synthetic materials for preparing hydrogels include polyethylene glycol (PEG), poly (Lactic-co-glycolic Acid) (PLGA), self-assembling peptide (SAP), tetronic-tyramine (Tet-TA), etc. (Figure 8). Polyethylene glycol (PEG) hydrogel has good biocompatibility, which is widely used in biomedical fields (Lin and Anseth, 2009). Mulyasasmita et al. (2014) prepared a protein-PEG hybrid hydrogel, which could co-deliver human induced pluripotent stem cell-derived endothelial cells (hiPSC-ECs) and VEGF. The *in vivo* results of a PAD mouse model revealed that the protein-PEG hydrogel could lower inflammation and decrease ischemia-reperfusion injury (Figure 9A). Poly (Latic-co-glycolic Acid) (PLGA) is also widely used in the preparation of biomaterials, and some researchers have made it into micron or nanoparticles and 3D scaffolds (Bharadwaz and Jayasuriya, 2020). In a recent study, Hong et al. (2020) prepared a PLGA-Cys-mPEG porous hydrogel and realized the “gel-solution” transition with glutathione (GSH). The hydrogel can not only support the formation of adipose-derived stem cells (ASCs) spheroids, but also treat hind limb ischemia by minimally invasive injection. The *in vivo* results indicated that the hydrogel had an apparent therapeutic effect in limb ischemia, which could enhance paracrine secretion of ASCs, promote angiogenesis of ischemic limbs (Figure 9B).

The development of self-assembling peptide (SAP) is inspired by the phenomenon of molecular self-assembly *in vivo*, such as protein winding, folding and the construction of DNA double helix (Palmer and Stupp, 2008). Huang et al. (2019a) synthesized a novel small molecular hydrogel by disulfide bond reduction, named Nap-GFFYK-Thiol hydrogel. The results showed that the hydrogel could not only improve the activity of human placental mesenchymal stem cells (hP-MSCs), but also promote blood flower recovery (Figure 9C). Since many research used synthetic hydrogels for LEAD, we summarized some of the studies in Table 3.

**TABLE 3:** Preclinical studies of synthetic hydrogels for LEAD.

Materials	Cargo	Animal	Injury model	Blood perfusion	Revascularization	Others	Refs.
PLGA-mPEG	ADSCs	Nude mice	Femoral artery and its branches ligation	Increased	Increased capillary and arteriole formation	Reduced tissue damage, increased the level of proangiogenic factors	Park et al. (2016)
Protein modified PEG	iPSC-ECs, VEGF	Nude mice	Femoral artery ligation	Not mentioned	Not mentioned	Induced tissue regeneration and decrease necrosis	Mulyasasmita et al. (2014)
PLGA-PEG-RADA peptide	VEGF, EPCs	Mice	Femoral artery ligation	Increased	Increased blood vessel density	Diminished inflammation, tissue fibrosis and degeneration	Li et al. (2017)
FN/Tet-TA copolymer	MSCs, ECs	Nude mice	Femoral artery and its branches excised	Not mentioned	Increased arteriole density	Reduced inflammation and tissue fibrosis	Jun et al. (2017)
RADA peptide	MSCs	Rats	Femoral artery excised	Increased	Not mentioned	Increased level of angiogenic markers, inhibited tissue apoptosis and fibrosis	Park et al. (2018)

#### 5.2.3 Hydrogel loaded with growth factors or stem cells

**FIGURE 10:**
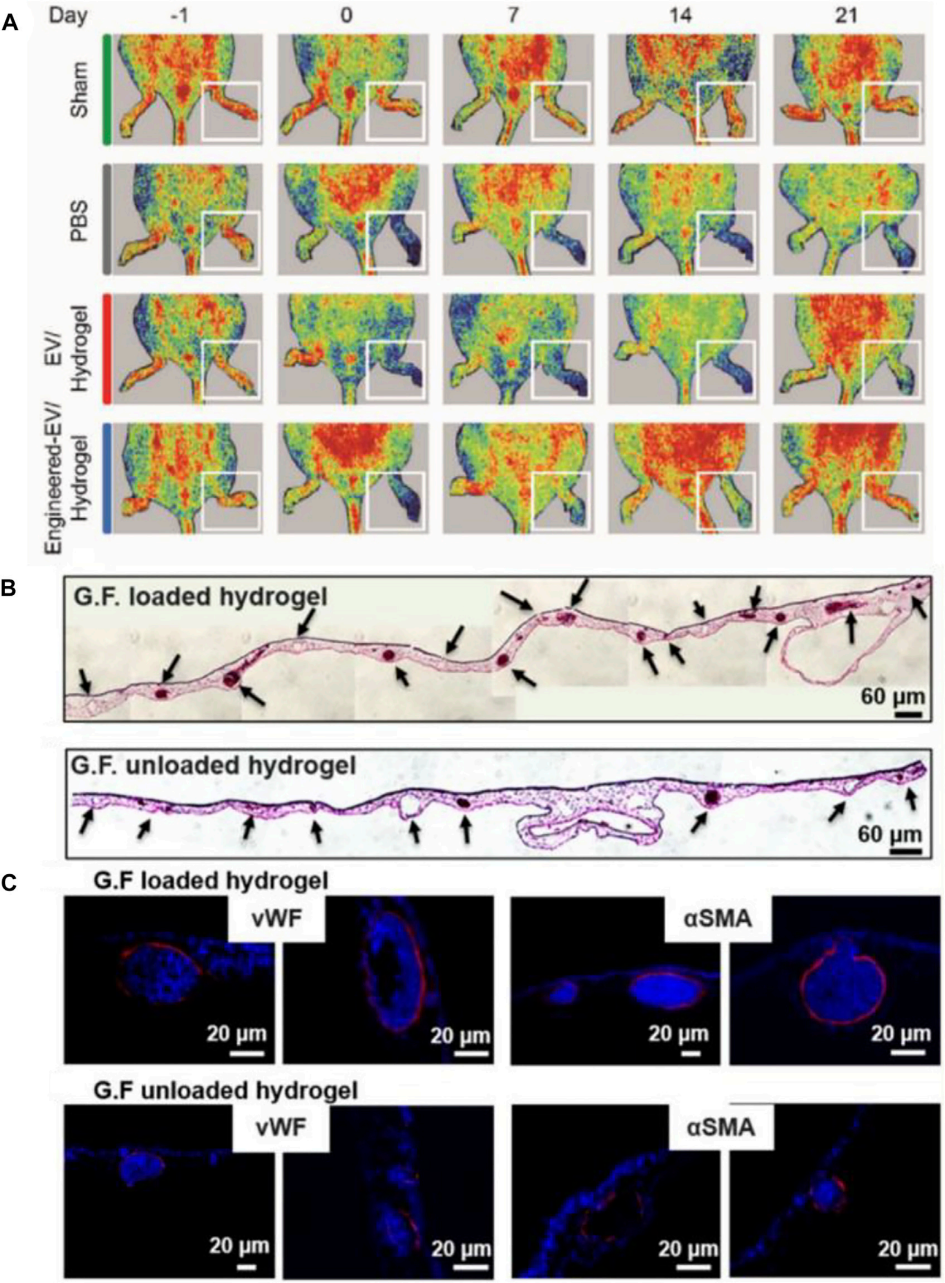
Schematic diagrams of hydrogels loaded with growth factors or stem cells to treat LEAD. **(A)** LDPI images of ischemic hind limbs in a mouse model showed that engineered-EV/hydrogel significantly improved blood perfusion and preserved the ischemic limb. **(B)** Representative images of H&E staining showed that the GF-loaded hydrogel could promote tube formation. Scale bar = 60 µm. **(C)** Representative images of immunohistochemistry staining showed that the GF-loaded hydrogel could improve angiogenesis. Scale bar = 20 µm. **(A)** is reprinted with permission from ref. (Xing et al., 2021b). **(B,C)** are reprinted with permission from ref. (Huang et al., 2019b).

With the boosting studies of biomaterials, more and more research is focusing on the combination of biomaterials with growth factors and stem cells. Among all these materials, hydrogel is frequently used due to its 3D porous structure and efficient water content. Thus, hydrogels are chosen as carriers for growth factors and stem cells to treat LEAD. In a recent study, Xing et al. (2021b) transfected VEGF and TFEB plasmids into extracellular vesicles (EVs) derived from endothelial cells. Then prepared a hydrogel loaded with VEGF/TFEB-engineered EVs. Their work demonstrated that the EV-loaded hydrogel could improve angiogenesis (Figure 10A). Huang et al. (2019b) designed a functionalized SAP hydrogel with RADA16-GGQQLK (QLK) and RADA16-GGLRKKLGKA (LRK) sequences. The LRK sequence is a cofactor that could help growth factors to encapsulate in this SAP hydrogel. The results revealed that the VEGF/HGF-loaded SAP hydrogel could promote tube formation *in vitro* and angiogenesis *in vivo* (Figures 10B,C). Stem cells therapy has been proved to treat limb ischemia, but it is still limited by problems such as inaccurate cell release and poor cell survival (Tang et al., 2017). Due to a lack of blood perfusion, the temperature of ischemic limb is often lower than normal tissues (Fernandez et al., 2018). Inspired by this, Wang et al. (2019) created a temperature-responsive hydrogel, which based on Methylcellulose (MC), encapsulated with human placental mesenchymal stem cells (PMSCs). The results showed that MC-based hydrogel can effectively release PMSCs into ischemic area, and then improve blood perfusion, enhance revascularization. The above experiments confirm that hydrogels as carriers have a good synergistic effect with growth factors or stem cells in promoting angiogenesis. Therefore, it is reasonable to believe that this type of research deserves to be further explored to facilitate the treatment of LEAD in the clinical field.

### 5.3 Other biomaterials

In addition to hydrogels, there are still many other types of biomaterials that have been studied to treat LEAD, such as scaffold (Li et al., 2018; Carrabba et al., 2020; Leclerc et al., 2021; Mu et al., 2021), nanoparticle (NP) (Matsumoto et al., 2020; Park et al., 2020; Liu et al., 2017; Albrecht-Schgoer et al., 2017) and spheroid (Park et al., 2014; Choi et al., 2021; Lee et al., 2021).

#### 5.3.1 Scaffold

**FIGURE 11:**
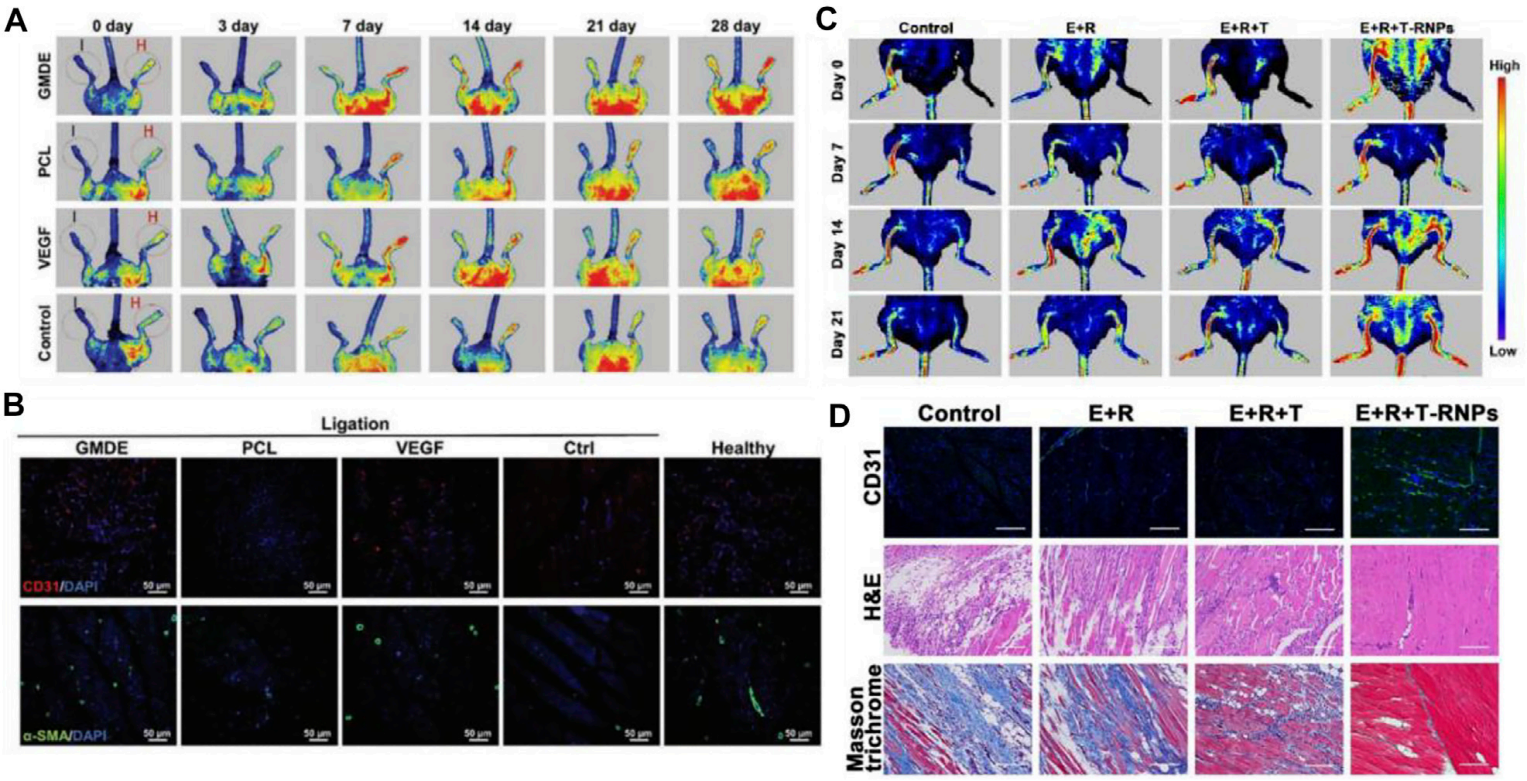
Schematic diagrams of scaffolds to improve blood perfusion. **(A)** LDPI images of ischemic hind limbs showed that GMDE scaffold group had the best angiogenic effect. **(B)** Immunofluorescent staining for CD31 and α-SMA of thigh muscle showed that GMDE scaffold could promote angiogenesis. Scale bar = 50 µm. **(C)** LDPI images of ischemic hind limbs showed that T-RNPs-anchoring hydrogel scaffold loaded with EPCs had the best angiogenic effect. **(D)** Immunofluorescent images of CD31, H&E, and Masson’s trichrome staining of ischemic muscles. The E + R + T-RNPs group had highest blood vessel density and lowest levels of inflammation and fibrosis. Scale bar = 200 μm. **(A,B)** are reprinted with permission from ref. (Mu et al., 2021). **(C,D)** are reprinted with permission from ref. (Li et al., 2018).

Many researchers have begun to focus on scaffold-based tissue engineering strategies to restore blood perfusion in ischemic limbs. Recently, Mu et al. (2021) fabricated an electrospun glucomannan decanoate (GMDE) scaffold to simulate microbial invasion and isolate endogenous galectin-1. The *in vivo* results found that GMDE scaffold can effectively promote blood perfusion (Figures 11A,B). Li et al. (2018) designed a nanoparticle-anchoring hydrogel scaffold and evaluated the therapeutic effect on ischemic hind limbs of mice. The *in vivo* results showed the scaffold could significantly promote the restoration of blood perfusion (Figures 11C,D).

#### 5.3.2 Nanoparticle

**FIGURE 12:**
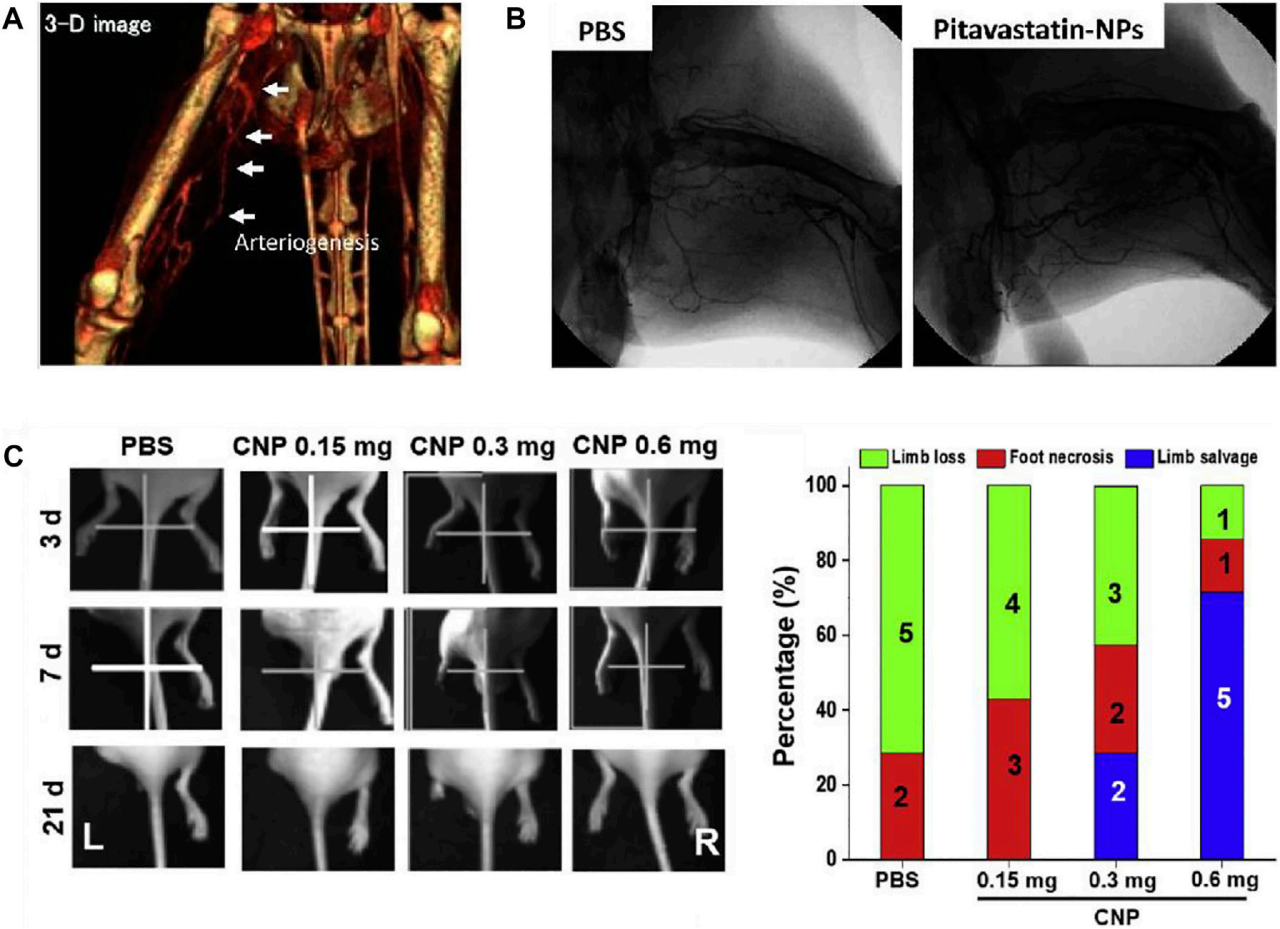
Schematic diagrams of nanoparticles to improve blood perfusion. **(A)** Representative angio-CT images demonstrated the development of collateral arteries. **(B)** Representative images of angiography of ischemic lower extremities. The pitavastain-NPs group showed the revascularization. **(C)** Representative images of limb loss in different treatment groups and quantification of limb loss, foot necrosis, and limb salvage. The CNP 0.6 mg group showed the best effect of limb salvage. **(A,B)** are reprinted with permission from ref. (Matsumoto et al., 2020). **(C)** is reprinted with permission from ref. (Park et al., 2020).

Nanoparticles (NPs) have emerged as a new model of drug delivery system with advantages such as targeting and slow release. Matsumoto et al. (2020) designed a NP-mediated, targeted drug delivery system using PLGA and statin as raw materials. The results revealed that Pitavastatin-NPs could significantly improve the therapeutic effect on limb ischemia (Figures 12A,B). According to many studies, cerium oxide nanoparticles (CNP) have shown therapeutic effects both *in vivo* (Naganuma and Traversa, 2014) and *in vitro* (Kwon et al., 2016). Park et al. (2020) confirmed the angiogenesis-promoting properties of CNP in a hind limb ischemia mouse model and further investigated molecular mechanisms by which it exerts its promoting effects. The results showed increased expression of pro-angiogenic markers, vascular maturation and muscle remodeling after intramuscular injection of CNP. Moreover, such an efficient pro-angiogenic effect may be due to the upregulation of the Ref-1/APE1 pathway (Figure 12C).

#### 5.3.3 Spheroid

**FIGURE 13:**
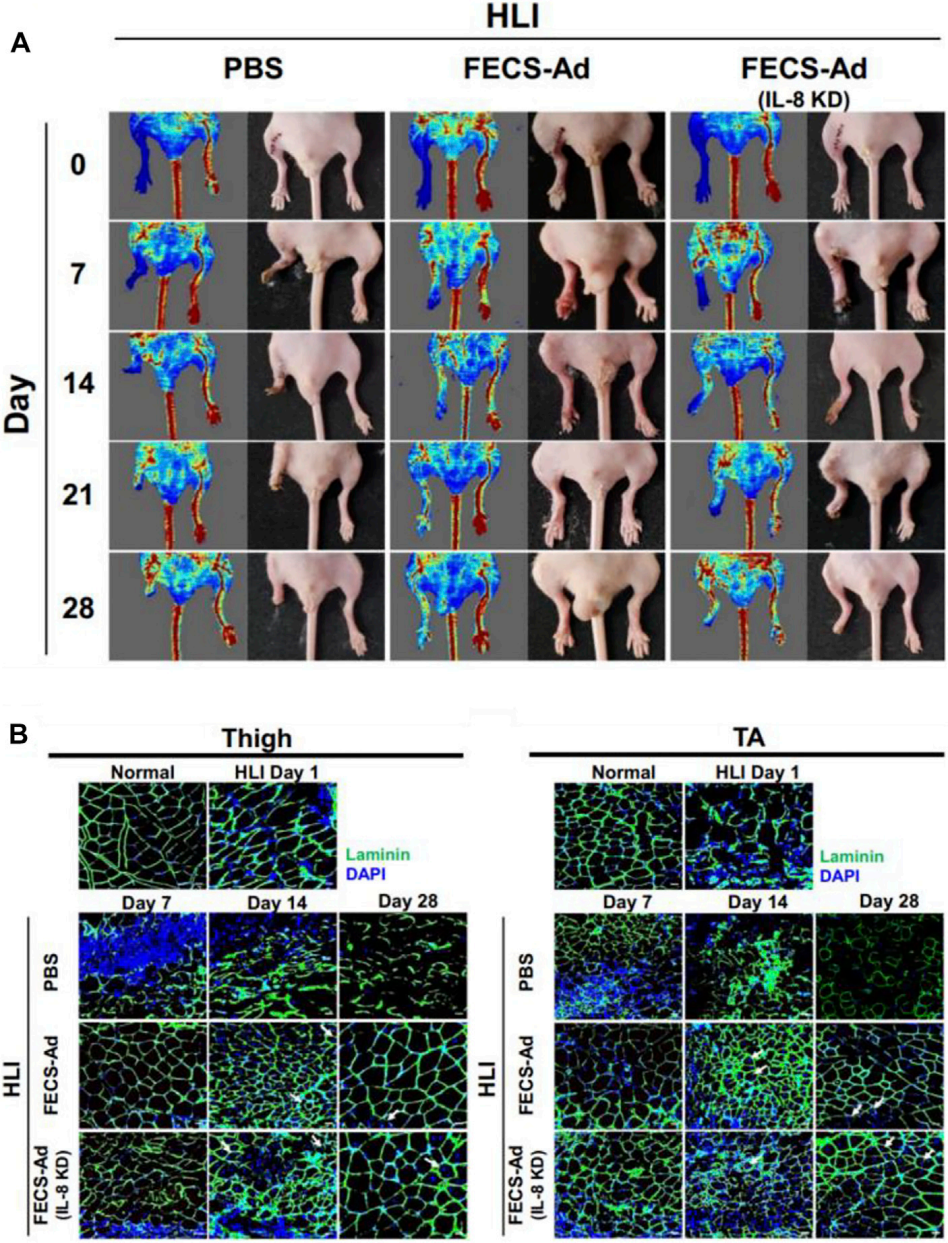
Schematic diagrams of spheroids to improve blood perfusion. **(A)** Representative images of LDPI and hind limb morphology. FECS-Ad treatment resulted in the best recovery of blood flow and it reduced limb loss. **(B)** Laminin staining of the thigh and TA muscles showed that FECS-Ad promotes muscle fiber regeneration and restores muscle structure. Scale bar = 20 μm. Figure adapted with permission from ref. (Choi et al., 2021).

As mentioned above, stem cell therapy in therapeutic angiogenesis is a reliable treatment for LEAD. The therapeutic benefit of single-cell transplantation is not ideal. Still, the efficiency will significantly enhance when cells become spheroids, because the 3D spheroid system could create a more biomimetic microenvironment (Lee et al., 2021). Therefore, cell spheroids that act synergistically with biomaterials have been extensively studied. Choi et al. (2021) cultured human adipose-derived stem cells (hASCs) in an FGF-2 matrix, which in turn developed into an enhanced cells spheroid (FECs-Ad) and were able to secrete a variety of angiogenic growth factors. Their work proved that the injection of FECs-Ad promotes angiogenesis in ischemic hind limbs, which also increases the expression of VEGF (Figures 13A,B).

Since there have been many studies using other biomaterials to investigate the treatment of LEAD, so we summarize some of the studies in Table 4.

**TABLE 4:** Preclinical studies of other biomaterials for LEAD.

Material forms	Materials	Cargo	Animal	Injury model	Blood perfusion	Revascularization	Others	Refs.
Nanoparticles	Dextran‐gelatin	VEGF	Mice	Femoral artery and its branches excised	Increased	Increased capillary density	Not mentioned	Xie et al. (2013)
Nanoparticles	Fragmine/protamine	bFGF	Rabbits	Femoral artery excised	Not mentioned	Increased collateral arteries	Not mentioned	Fujita et al. (2013)
Nanoparticles	Fragmine/protamine	bFGF	Mice	Femoral artery excised	Increased	Increased arterioles	Limb salvage and improved oxygen reperfusion	Nakamura et al. (2012)
Nanoparticles	PLGA	bFGF	Mice	Occlusion of the saphenous artery	Not mentioned	Increased number and diameter of arterioles	Not mentioned	Chappell et al. (2008)
Microspheres	Dextran‐PLGA	VEGF	Rats	Femoral artery excised	Not mentioned	Increased capillary and arteriole density	Enhanced the proliferation of endothelial cells	Zhang et al. (2019)
Microspheres	Gelatin	bFGF	Dogs	Iliac artery ligation and femoral artery excised	Increased	Increased capillary and arteriole density	Not mentioned	Zhao et al. (2011)
Porous scaffold	Collagen	BMMSCs	Rabbits	Femoral artery excised	Increased	Increased capillary and arteriole density	Increased oxygen saturation ratio	Wang et al. (2012)
Electrospun scaffold	Polycaprolactone/gelatin scaffolds	iPSCs‐ECs	Mice	Subcutaneous	Increased	Increased capillary and arteriole density	Increased VEGF expression and reduction of the inflammatory	Tan et al. (2018)

## 6 Conclusion and future perspectives

As diabetes continues to increase around the world, complication also arises. In all vascular complications of diabetes, LEAD is an essential cause of death for patients. The end-stage of LEAD is called CLTI, and the study found that the 5-years survival rate of diabetic patients with CLTI is lower than those without limb ischemia. Therefore, they need more effective treatments to repair ischemic limbs, minimize amputation and mortality. Conventional treatments include medication, open surgery, endovascular treatment, amputation and lifestyle management are not always enough. Crucial aspect is the lack of awareness of the disease among both physicians and patients. This means that patients often present to the physician’s attention already with an advanced disease: CLTI. In these cases, currently available treatment options often perform poorly. The therapeutic angiogenesis strategy proposed in recent years aims to promote the spontaneous formation of revascularization and restore the blood perfusion of ischemic limbs through growth factors and stem cell transplantation. However, all of the above treatments have their limitations, urging us to explore novel methods to treat diabetic LEAD. With the continuous development of biomedicine and tissue engineering, many natural or synthetic biomaterials have been used to research of limb ischemia mouse models due to their promising biological properties. At present, many researchers have confirmed the effectiveness of biomaterials to rescue limb ischemia. Unfortunately, most of the studies are still in the experimental animal stage, and the animal models established are not as complicated as many other complications of clinical diabetic patients. Although some clinical trials have already verified the safety of the biomaterials they use, however, the translation of biomaterials into clinical products still needs to face many questions. For example, whether biomaterial-based therapeutic angiogenesis strategies can produce good angiogenesis-promoting effects in real-world patients is still debatable; and how to translate various biomaterials from small-scale application in the laboratory stage to high-volume use in treating patients in the clinic. Therefore, randomized clinical trials are needed to clarify the role of these new technologies in treating patients with diabetic LEAD. In conclusion, we hope that biomaterials will be further developed and gradually applied to clinical treatment from the current animal research stage, thus providing more ways to treat diabetic LEAD.

## Glossary

ABI: ankle-brachial index
ACEIs: angiotensin-converting enzyme inhibitors
ADSCs: adipose-derived stem cells
AGEs: advanced glycation end products
apoB: apolipoprotein B
ARBs: angiotensin receptor antagonists
bFGF/FGF-2: basic fibroblast growth factor
BMMNCs: bone marrow mononuclear cells
BMMSCs: bone marrow mesenchymal stem cells
BMSCs: bone marrow stem cells
CLTI: chronic limb threatening ischemia
CNP: cerium oxide nanoparticle
CSCs: cardiac stem cells
CV: cardiovascular
CVD: cardiovascular disease
DFU: diabetic foot ulcer
DPP-4is: dipeptidyl peptidase-4 inhibitors
E + R + T-RNPs: EPCs + RADA16 + tacrolimus-loaded RADA16 peptide-modified nanoparticles
ECM: extracellular matrix
ECs: endothelial cells
EGF: epidermal growth factor
EGFP: enhanced green fluorescent protein
EGM-2: endothelial growth medium-2
EMCV: encephalomyocarditis virus
EPCs: endothelial progenitor cells
EPO: erythropoietin
EVs: extracellular vesicles
EVT: endovascular treatment
FA: ferulic acid
FECS-Ad: functionally enhanced cells spheroid
FGF: fibroblast growth factor
FN: fibronectin
G-CSF: granulocyte-colony stimulating factor
GFs: growth factors
GLP-1RAs: glucagon-like peptide-1 receptor agonists
GMDE: glucomannan decanoate
GSH: glutathione
hASCs: human adipose-derived stem cells
HbA1c: haemoglobin A1c
HGF: hepatocyte growth factor
HIF-1α: hypoxia-inducible factor-1α
HLI: hindlimb ischemia
HUVECs: human umbilical vein endothelial cells
IGF: insulin-like growth factor
IL-1a: interleukin-1a
iPSCs-ECs: induced pluripotent stem cells-endothelial cells
LDL-C: low-density lipoprotein cholesterol
LDPI: laser doppler perfusion imaging
LEAD: lower extremity arterial disease
MACE: major adverse cardiovascular event
MALE: major adverse limb event
mPEG: modified polyethylene glycol
MSCs: mesenchymal stem cells 5
NO: nitric oxide
non-HDL-C: non-high-density lipoprotein cholesterol
NPs: nanoparticles
PAD: peripheral arterial disease
PBMNCs: peripheral blood mononuclear cells
PBSCs: peripheral blood stem cells
PCL: polycaprolactone
PCSK9: proprotein convertase subtilisin/kexin type 9
PDGF: platelet derived growth factor
PGE1: prostaglandin E1
PLGA: poly (lactic-co-glycolic Acid)
SEM: scanning electron microscope
SGLT2is: sodium glucose co-transporter 2 inhibitors
T2DM: type 2 diabetes mellitus
TA: tibialis anterior
TBI: toe-brachial index
TcPO2: transcutaneous oxygen pressure
Tet-TA: tetronic-tyramine
TNF-α: tumor necrosis factor-α
VEGF: vascular endothelial growth factor
vWF: von Willebrand factor
α-SMA: α-Smooth muscle actin
